# Supplementary material for: Sex differences in social support perceived by polymedicated older adults with multimorbidity. MULTIPAP study
Source: PLoS One. 2022 Jul 27;17(7):e0268218. doi: 10.1371/journal.pone.0268218 (PMC9328549; doi:10.1371/journal.pone.0268218)
Supplement: Attachment — Submitted filename: Response to Reviewers.docx [file pone.0268218.s001.docx]

**Response to Reviewers:**

***Reviewer #1: This manuscript uses survey data from Spanish health centers to assess the sex differences in social support among older polymedicated adults. Overall, it is fairly well written and easy to follow. Please see below for my comments.***

***1. For the most part, the authors do well to use politically correct language when referring to older adults. However, on line 45, per the Gerontological Society of America’s policy on language regarding the older population, I suggest replacing the term ‘elderly people’ with a more neutral term (e.g., older adults, older people). See for (Lundebjerg et al., 2017) for details on this policy.***

Following the reviewer's indications, we have checked the document and replaced “elderly people” by “older adults”. We appreciate the reference provided on this policy.

**2. The authors do well to distinguish between social networks and social support (lines 56-65). However, after introducing the concept of networks (including size, density, homogeneity, etc) in the Introduction, it was a little disappointing to see structural social support later operationalized simply as marital status and number of cohabitants in the home (lines 122-123). While these measures are often indicative of social support, their mere presence does not automatically guarantee social support. For instance, poor marriages might actually cause more strain than support (de Jong Gierveld et al. 2009). I realize that data limitations are always going to be an issue, but it seems that it is at least acknowledging the lack of social network data and marital quality as limitations in this study.**

We would appreciate this reflection.

The original version of the manuscript integrated the limitations into the discussion section. Following your suggestions, we have incorporated a separate heading called "Limitations and Strengths". The text we have reflected is as follows:

“The study of social support presents difficulties in relation to conceptualisation and measurement. In this study, structural social support has been studied through a proxy of the social network such as marital status and the number of cohabitants in the household. This is undoubtedly a limitation, since the structural perspective studies social networks, including all the individual's contacts and providing information on their dimensions, and not only the variables available in this research. However, despite this limitation, this proxy allows us to cover part of the structural social support, following the recommendations of the authors who state that it is more appropriate to study both types of support, functional and structural”.

**3. In the Materials and Methods section, the authors choose to separate functional support into ‘confident support” and “affective support” (lines 131-133). If they are going to make this distinction methodologically, it should be preceded with conceptual justification in the Introduction section.**

We are grateful for this note. We agree that including this information in the Introduction will help to better understand this distinction. We have included in the Introduction section an explanation of the two-dimensionality of social support as confidential and affective: “One of the most widely used instruments to study perceived functional social support is the Duke-Unk-11, Functional Social Support questionnaire (DUFSS) developed by Broadhead et al. [1]. It originally consisted of 14 items that Broadhead reduced to 11 items in its first validation, and after factor analysis the two-dimensionality of the questionnaire was confirmed. It was identified that on the one hand it measures "confidential support" (the possibility of having people to communicate with) and on the other hand it measures "affective support" (demonstrations of love, affection and empathy)”.

**4. I don’t understand the values given in lines 175-178). They do not seem to match those in Table 3. The bivariate comparisons given in the text do not make sense to me because they are not dichotomous outcomes. Rather there appears to be three response categories to each outcome.**

Thank you for highlighting this. We agree with the reviewer in providing the text results by columns, as in Table 3. We have made the suggested change.

**5. I am confused by the sentence on line 215 that reads “The increasing feminization of old age has meant that widowhood is a mostly female experience.” What does this mean? Are the authors trying to say that women are more likely to experience widowhood because they tend to live longer than men?**

This is a good consideration, thank you.

According to data from the last quarter of 2020 from the National Statistics Institute (INE) [2], the total number of widows and widowers in Spain amounts to 2.917.800 people. Of these, 2.331.200 are women, while men account for only 586.600. In other words, 80% of widowers in Spain are women. With regard to age, the statistics confirm that the situation of widowhood is directly related to age: the older you are, the greater the possibility of being widowed. The greater longevity of women compared to men explains why the number of women in this situation is much higher than that of men. Thus, the average age of Spanish widowers is 73, while that of women is 77.

**6. The sentence on lines 241-243 the authors say that their heterogeneous sample “is representative of the general multimorbid and polymedicated population, increasing its external validity.” But it seems to me that the external validity depends more on the sampling methodology (i.e., random sampling drawn from a representative sampling frame) rather than the sample demographics. It is difficult to tell if participants were randomly selected or if a convenience sampling method was used instead. Perhaps more importantly, however, the participants appear to have first been contacted in health centers, which—if true—would not make it a representative sample of the general population since only those who went to the health centers were eligible for being selected into the study. These distinctions should be noted in the manuscript.**

Thank you for this comment. The data used in this study are the baseline data from a randomised clinical trial, MULTIPAP STUDY [3]. It is a pragmatic cluster randomised controlled clinical trial with 12 months follow-up. The unit of randomisation was the family doctor and the unit of analysis was the patient. Although it cannot be said that this is a representative sample of the population, it is very close because in Spain, in 5 years, 90% of the population has consulted primary care. This is because the Spanish National Health System guarantees universal coverage and free access to health care for all Spanish nationals, regardless of their economic situation or their participation in the social security network. Primary care is the gateway to the health system where people with multimorbidity and polypharmacy regularly receive comprehensive, continuous and coordinated care for their health and funded prescription of medicines.

The full design of the MULTIPAP STUDY is described in the published protocol [3]. A brief description is included in the methodology section.

**Reviewer #2: I have just read the manuscript entitled "Sex differences in social support perceived by polymedicated older adults with multimorbidity. MULTIPAP Project", an interesting work exploring determinants of social support perceived by sex. The study is focused on polymedicated older adults with multimorbidity. After reading the manuscript, I have the following comments:**

**- The article is focused in older adults, although only in those aged 65-74 years. Why people older than 75 were not considered in the sample?**

Thank you for your encouragement us to give more details.

A comprehensive follow-up protocol for polimedicated patients aged 75 and over has been well established in Spain since many years. This is the reason why we excluded this age group in order to not interfere with the ongoing health care strategies. The population aged 74 and below, however, is not subjected to any specific control or prevention strategies regarding polimedication. Moreover, the age group known as young-old patients (65-74 years) presents relatively high rates of multimorbidity and polypharmacy, representing an age group in which there is room for improvement, and which could benefit from interventions aimed at improving the adequacy of their prescriptions and prevention of drug-related harms. Results from the EpiChron Cohort, one of the Spanish cohort leaders by researches of our group, showed that more than 70% of people aged 60-74 have multimorbidity and around 70% of people aged 65+ are prescribed with polypharmacy.

A short note on this has been included in the Introduction section.

**- All the instruments used in this study have been previously validated in other samples. I wonder whether the authors could provide data on internal consistency (e.g., Cronbach’s alpha for unidimensional scales) according to the sample considered in this study.**

The requested data have been included in the Material and Methods section.

**- In Table 4, the 95% CI associated to “Number of drugs” in men cannot provide a significant p-value. This would be checked and also revised in the Results section in the main text.**

Thank you for this indication, there was a mistake in the symbol. To avoid reading difficulties, we have replaced the minus symbol with inverted commas and checked the text in the Results section.

**- The authors have found an association between lower perception of social support and multimorbidity in men. This result is not observed in women and should be discussed.**

Thank you for the opportunity to review it. We have found that the association between lower perceived social support and multimorbidity in men is not a statistically significant result. We have removed this sentence from the results section.

**- Potential limitations of this study should be highlighted in the Discussion section.**

The original version of the manuscript integrated the limitations into the discussion section. Following the suggestions of both reviewers, we have incorporated a separate heading called "Limitations and Strengths".

**Minor comments:**

**- Line 101: "Random sampling". Which type of random sampling was used?**

Thank you for this comment. The data used in this study are the baseline data from a randomised clinical trial, the MULTIPAP STUDY [3]. It is a pragmatic cluster randomised controlled clinical trial with 12 months of follow-up. Patients were added to a randomised list of potential participants, provided they met the inclusion criteria.

The unit of randomisation was the family doctor and the unit of analysis was the patient. Randomisation of the family doctor was performed using the treatment allocation module of the Epidat 4.1 programme.

**- Lines 141-142: “Explanatory linear regression models”. Why explanatory?**

Thank you for giving us the opportunity to clarify the final model. An explanatory model was chosen since the main objective was to estimate the effect of sex on functional social support adjusted for the other variables.

**- Lines 190-192: “In contrast, a higher number of diseases was associated with a lower social support score; it fell by 0.4 points (95% CI -0.87–0.30) for each disease”. Please revise the use of hyphen in confidence intervals when negative values are reported. On the other hand, it is not a significant result.**

We agree, it is not a statistically significant result. We remove that sentence from the results section.

References

*1.	Broadhead WE, Gehlbach SH, de Gruy F V, Kaplan BH. The Duke-UNC Functional Social Support Questionnaire. Measurement of social support in family medicine patients. Med Care. 1988;26: 709–23. Available: http://www.ncbi.nlm.nih.gov/pubmed/3393031*

*2.	Instituto Nacional de Estadística (INe). Resultados Nacionales. Población de 16 y más años por estado civil, sexo y grupo de edad [Internet]. Resultados nacionales. 2021. Available: https://www.ine.es/jaxiT3/Datos.htm?t=4031#!tabs-grafico*

*3.	Prados-Torres A, del Cura-González I, Prados-Torres D, López-Rodríguez JA, Leiva-Fernández F, Calderón-Larrañaga A, et al. Effectiveness of an intervention for improving drug prescription in primary care patients with multimorbidity and polypharmacy: study protocol of a cluster randomized clinical trial (Multi-PAP project). Implement Sci. 2017;12: 54. doi:10.1186/s13012-017-0584-x*
